# Supplementary figures and images for: Master corepressor inactivation through multivalent SLiM-induced polymerization mediated by the oncogene suppressor RAI2
Source: Nat Commun. 2024 Jun 19;15:5241. doi: 10.1038/s41467-024-49488-3 (PMC11187106; doi:10.1038/s41467-024-49488-3)

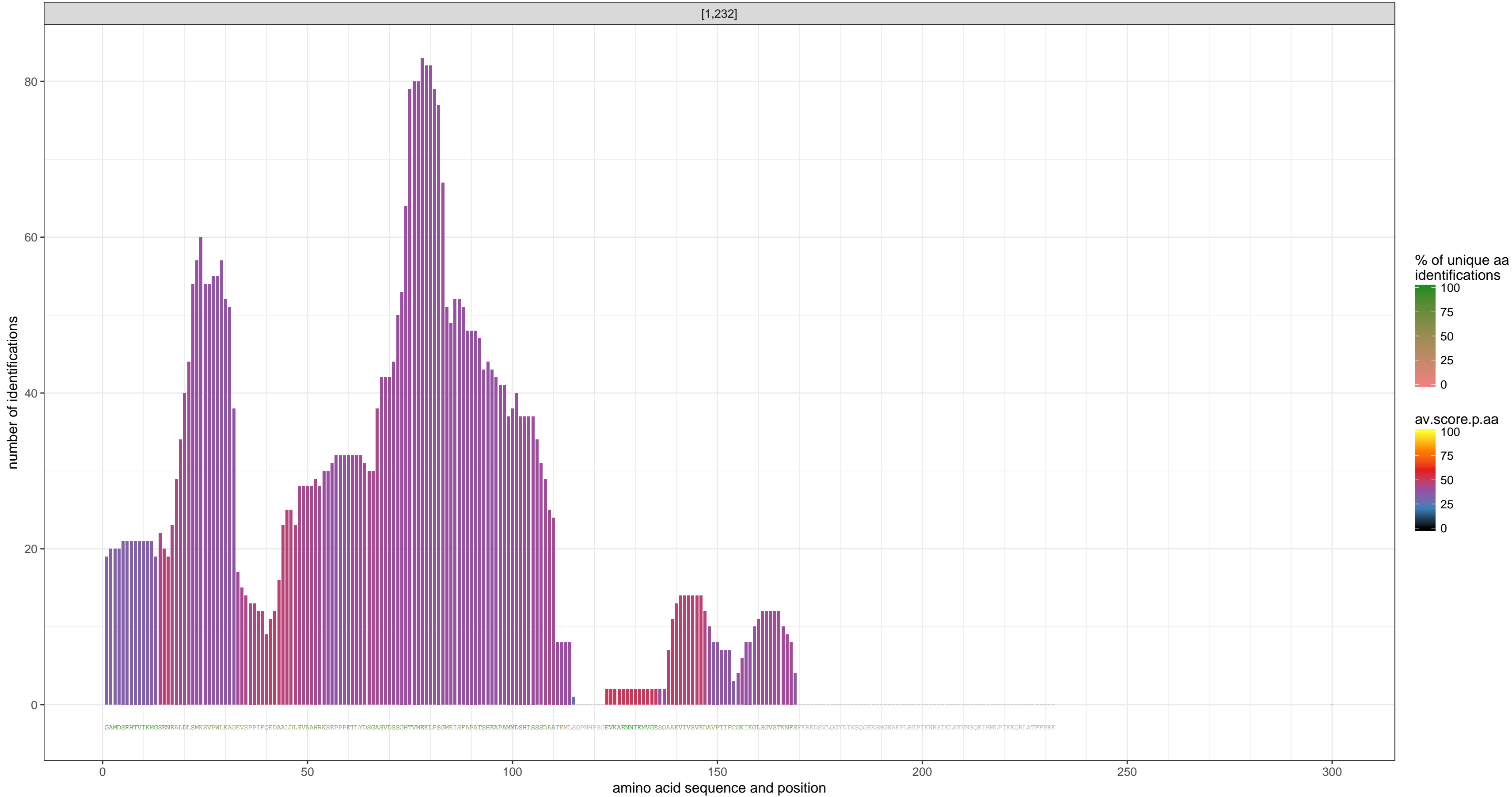

180222\_band02\_R1

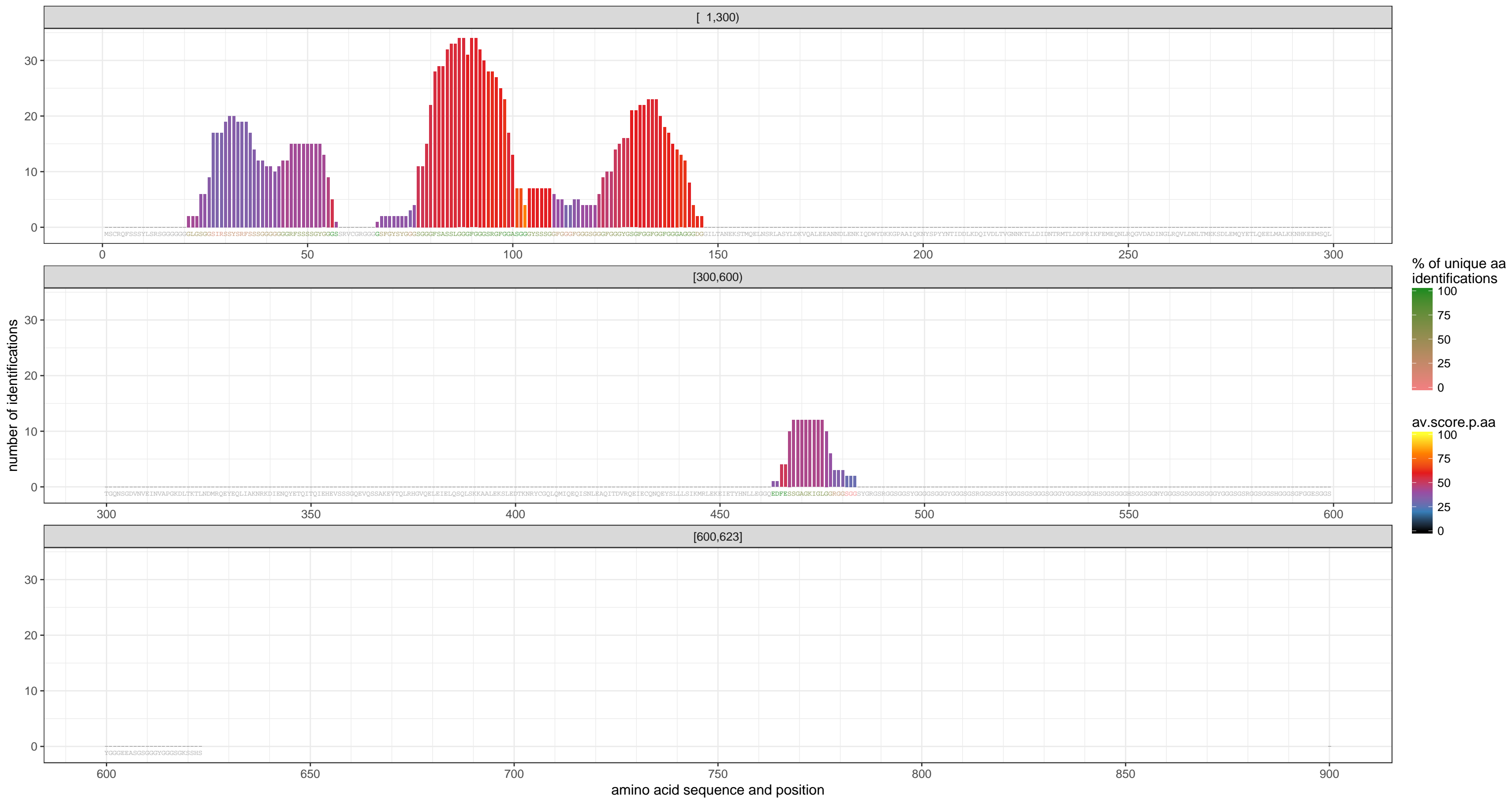

180222\_band02\_R1

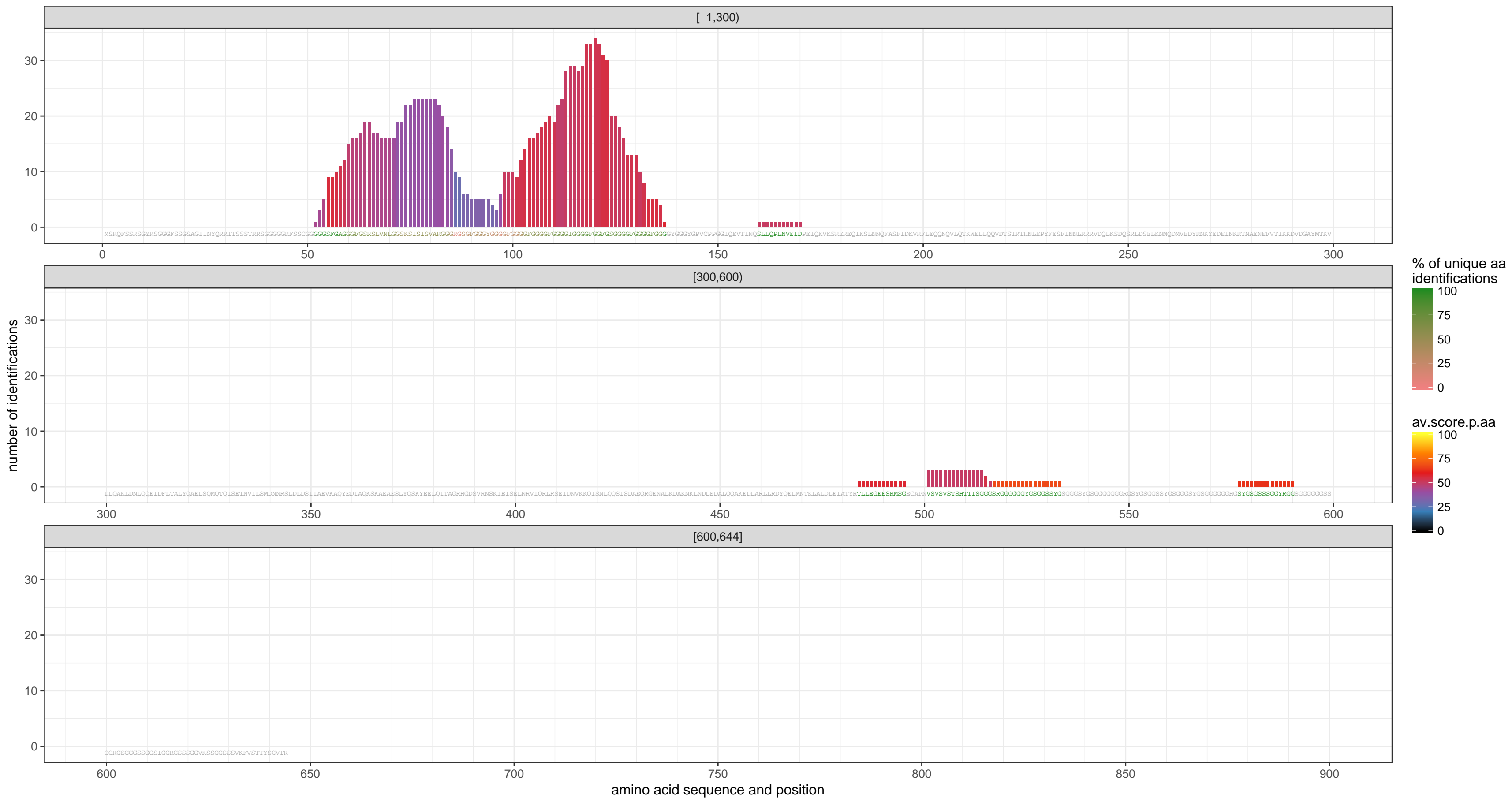

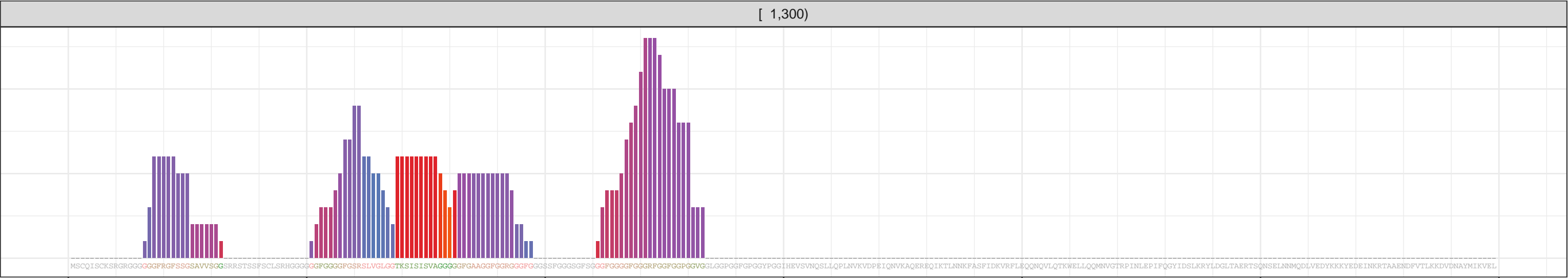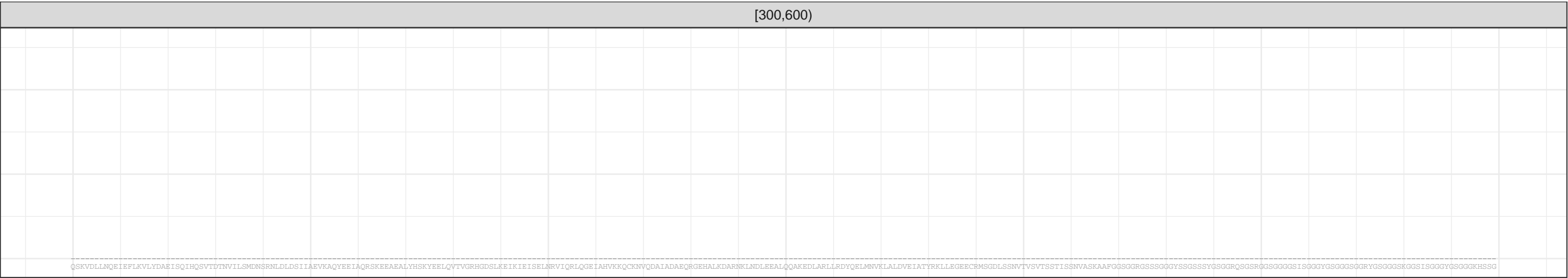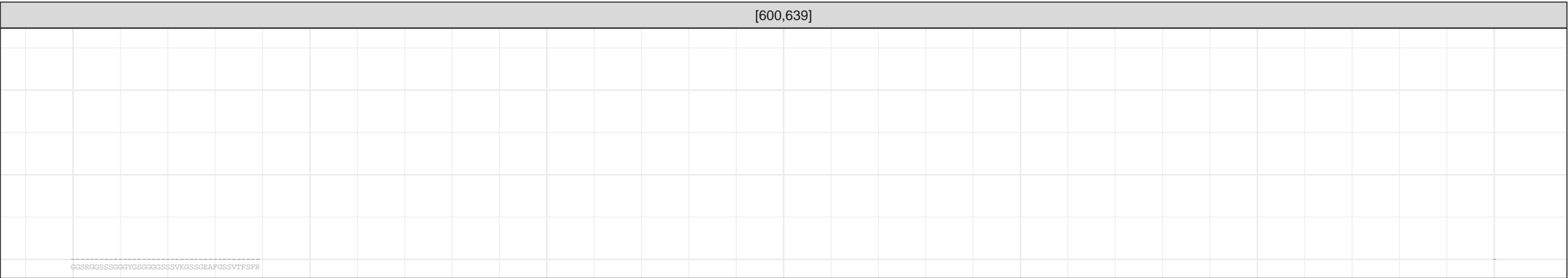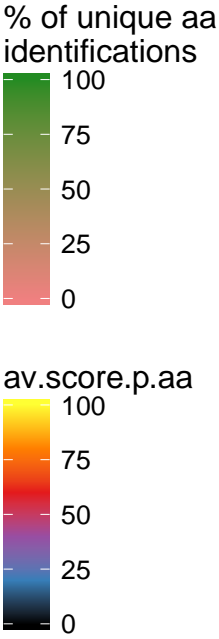

180222\_band02\_R1

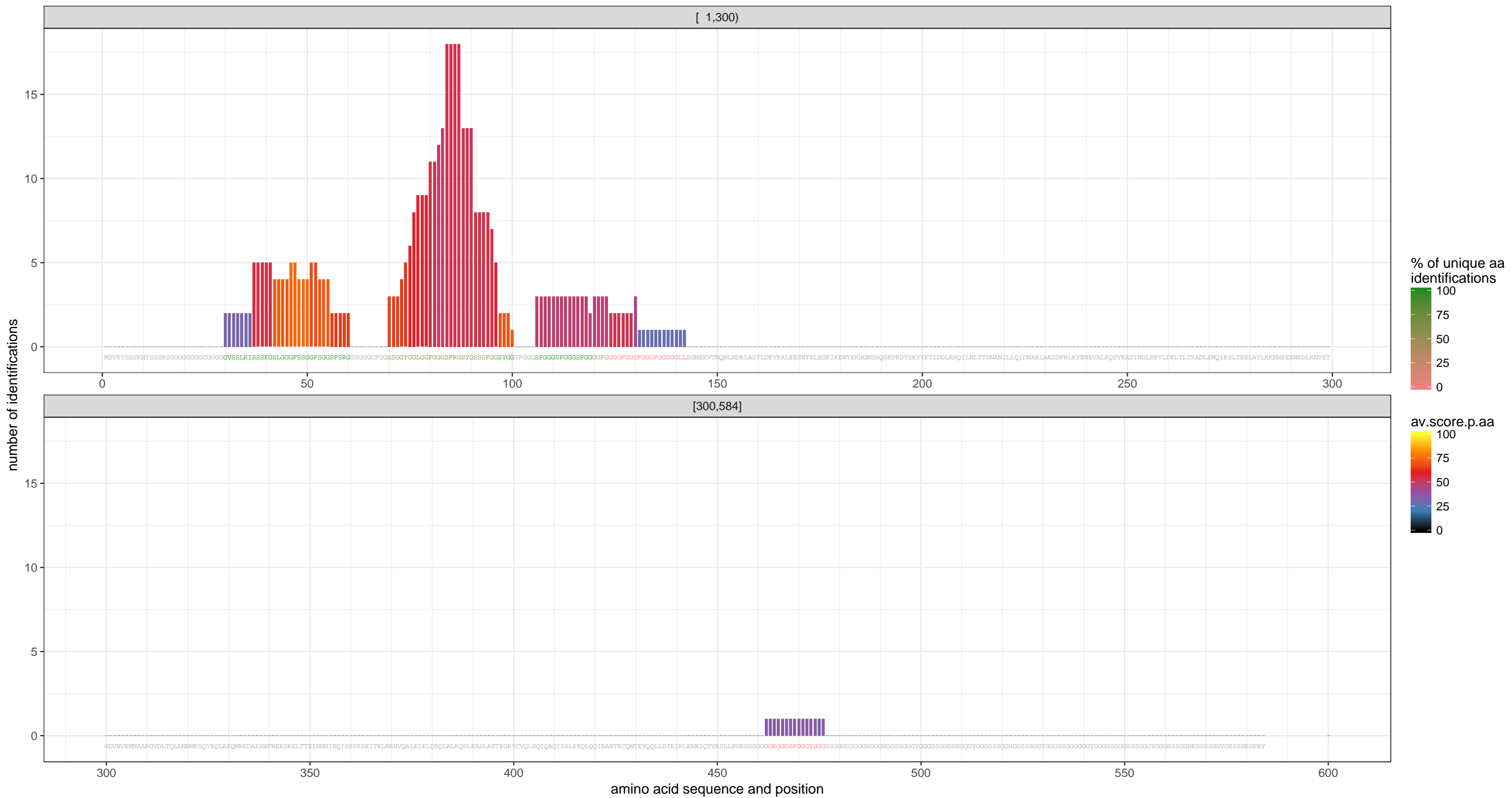

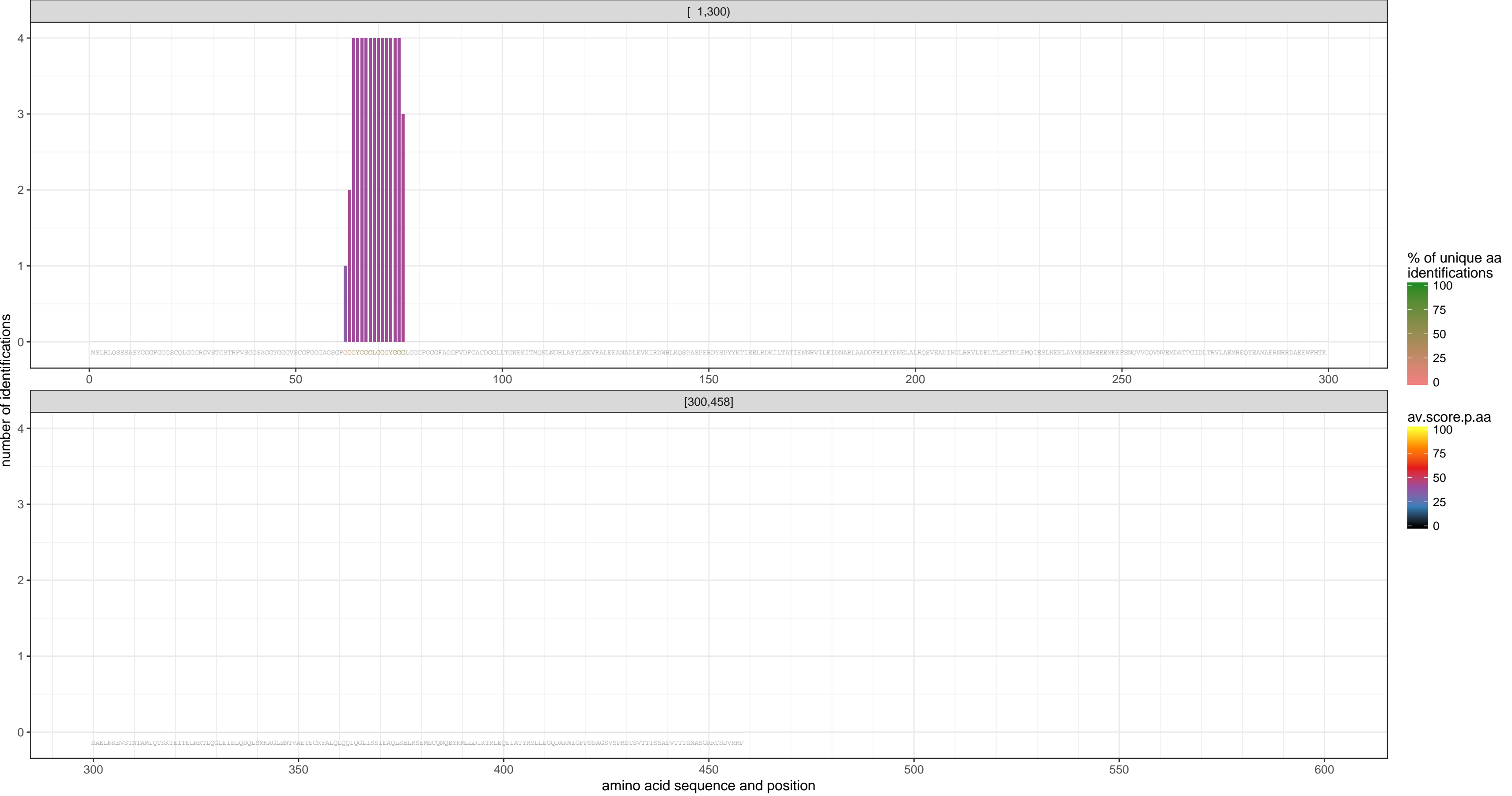

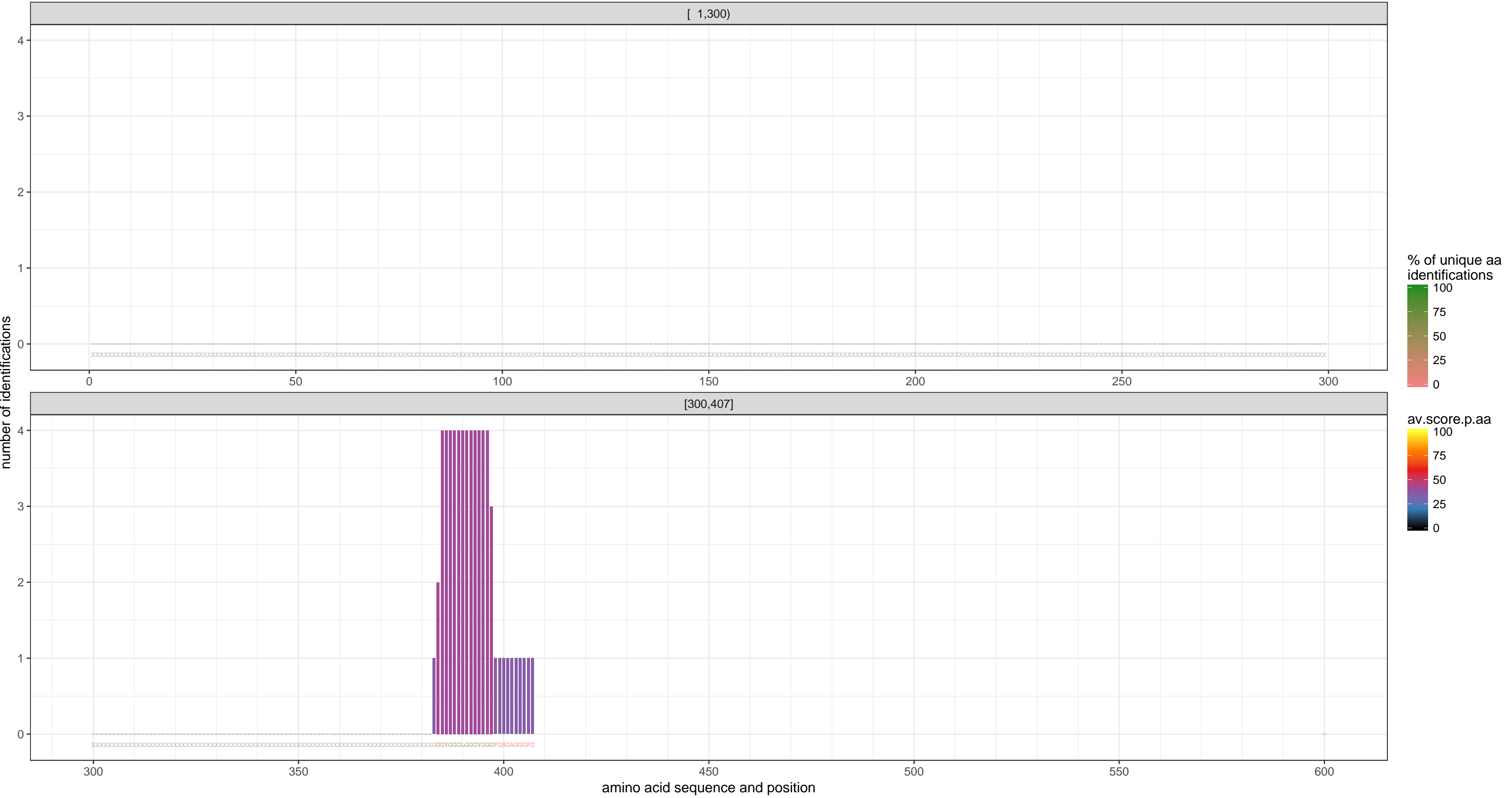

– protein\_id: ###REV#####CONTAMINANT###P13645 – sequence coverage: 2.8 % – total score: 220

180222\_band02\_R1

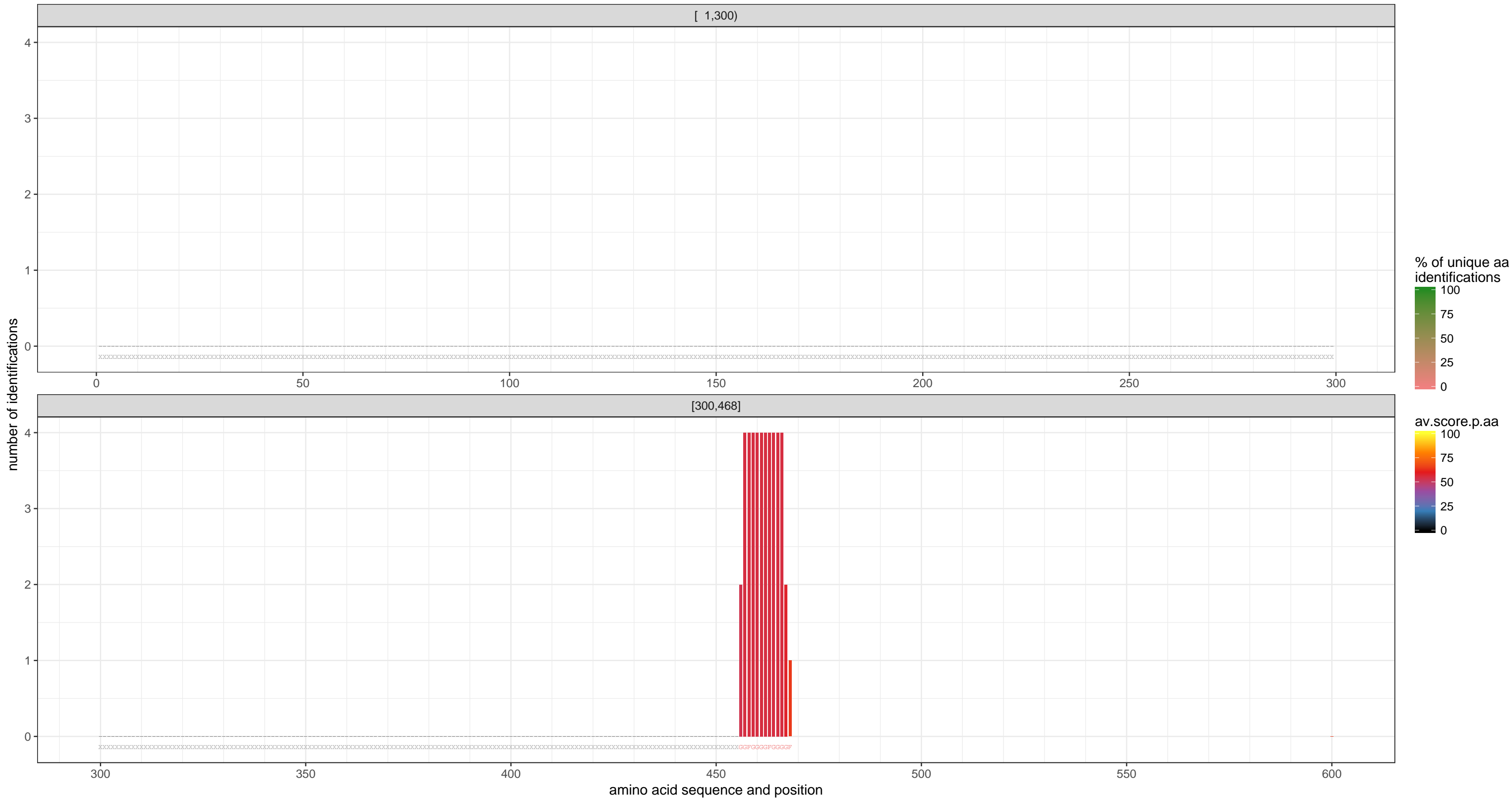

180222\_band02\_R1

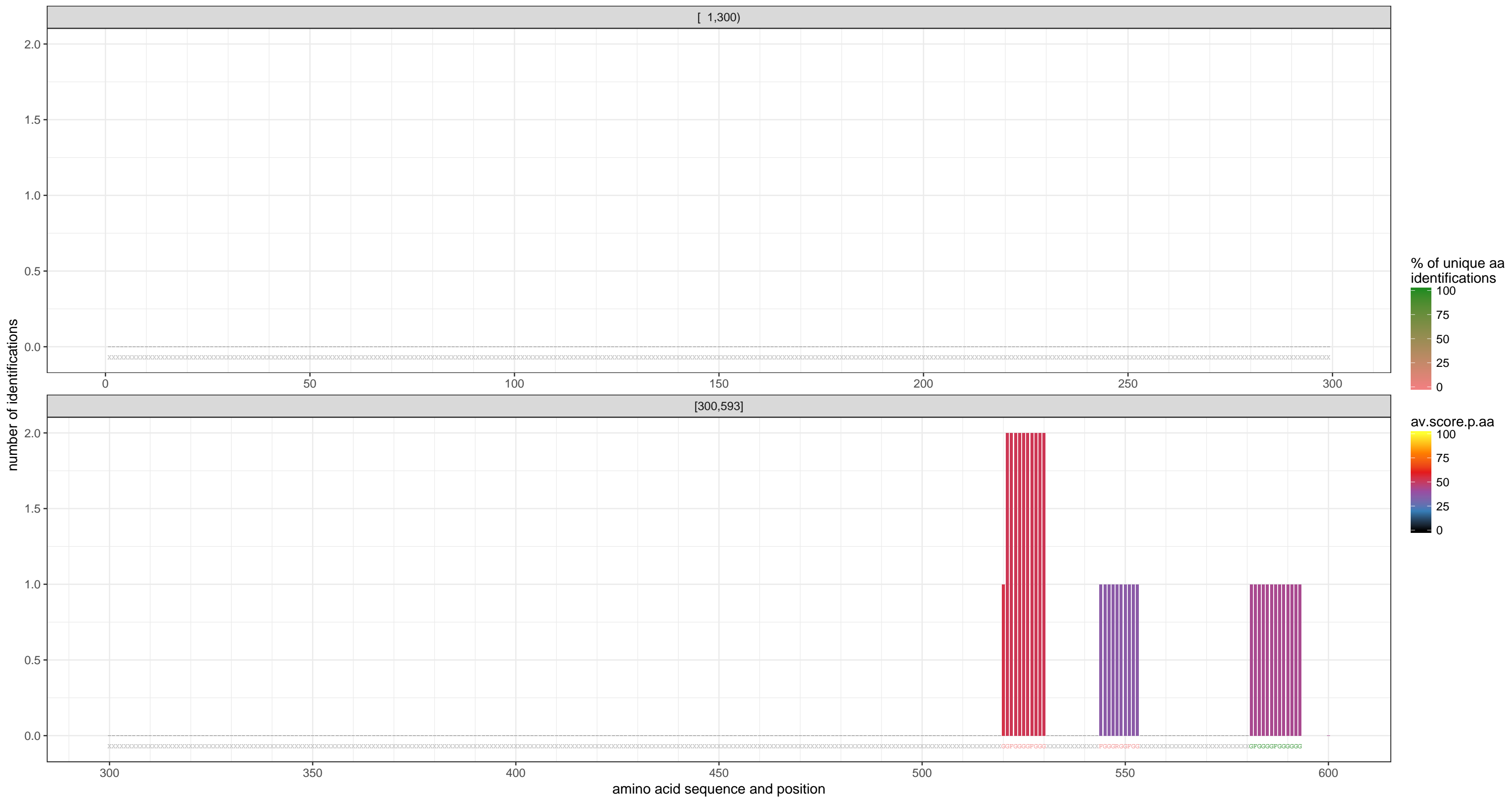

Supplement: Supplementary file 11 — Supplementary Data 8 [file 41467_2024_49488_MOESM11_ESM.pdf]
