# Supplementary material for: Master corepressor inactivation through multivalent SLiM-induced polymerization mediated by the oncogene suppressor RAI2
Source: Nat Commun. 2024 Jun 19;15:5241. doi: 10.1038/s41467-024-49488-3 (PMC11187106; doi:10.1038/s41467-024-49488-3)
Supplement: Supplementary file 12 — Reporting Summary [file 41467_2024_49488_MOESM12_ESM.pdf]

nature portfolio

Corresponding author(s): Klaus Pantel, Matthias Wilmanns

Last updated by author(s): 27 May, 2024

Reporting Summary

Nature Portfolio wishes to improve the reproducibility of the work that we publish. This form provides structure for consistency and transparency in reporting. For further information on Nature Portfolio policies, see our Editorial Policies and the Editorial Policy Checklist.

Statistics

For all statistical analyses, confirm that the following items are present in the figure legend, table legend, main text, or Methods section.

n/a	Confirmed	
☐	☒	The exact sample size (*n*) for each experimental group/condition, given as a discrete number and unit of measurement
☐	☒	A statement on whether measurements were taken from distinct samples or whether the same sample was measured repeatedly
☐	☒	The statistical test(s) used AND whether they are one- or two-sided<br>*Only common tests should be described solely by name; describe more complex techniques in the Methods section.*
☒	☐	A description of all covariates tested
☒	☐	A description of any assumptions or corrections, such as tests of normality and adjustment for multiple comparisons
☐	☒	A full description of the statistical parameters including central tendency (e.g. means) or other basic estimates (e.g. regression coefficient) AND variation (e.g. standard deviation) or associated estimates of uncertainty (e.g. confidence intervals)
☐	☒	For null hypothesis testing, the test statistic (e.g. *F*, *t*, *r*) with confidence intervals, effect sizes, degrees of freedom and *P* value noted<br>*Give P values as exact values whenever suitable.*
☒	☐	For Bayesian analysis, information on the choice of priors and Markov chain Monte Carlo settings
☒	☐	For hierarchical and complex designs, identification of the appropriate level for tests and full reporting of outcomes
☒	☐	Estimates of effect sizes (e.g. Cohen's *d*, Pearson's *r*), indicating how they were calculated

Our web collection on statistics for biologists contains articles on many of the points above.

Software and code

Policy information about availability of computer code

Data collection

Mass Spectrometry: Orbitrap Fusion Lumos mass spectrometer (Thermofischer Scientific, MA, USA)
Quantitative determination of binding affinities: VP-ITC system (Malvern Pananalytical, UK)
Analytical size exclusion chromatography: Superose 6 3.2/300 GL column (Cytiva, MA, USA) coupled to a 1260 Infinity HPLC system (Agilent, CA, USA)
Circular dichroism spectropolarimetry: Chirascan CD spectrometer (Applied Photophysics, UK)
Small Angle X-ray Scattering: beamline P12 at PETRA III (EMBL/DESY, Hamburg, Germany)
X-ray structure determination: beamline P13 at PETRA III (EMBL/DESY, Hamburg, Germany)
NMR spectroscopy: 600 MHz NMR spectrometer (Bruker, MA, USA), 700 MHz Avance III spectrometer (Bruker, MA, USA)
Negative-stain electron microscopy: Talos L120C transmission electron microscope equipped with a 4K Ceta CEMOS camera using TIA 4.1.5 (Thermo Fisher Scientific, MA, US).
Cryogenic-EM: Titan Krios electron microscope operating at 300 kV and equipped with a K3 direct electron detector (Gatan, CA, USA) using a GIF quantum energy filter with a 20-eV slit width (Thermo Fisher Scientific, MA, USA).
Immunofluorescence staining: Leica TCS SP5 microscope (Leica Microsystems, Germany)

Data analysis

Mass Spectrometry: IsobarQuant, https://github.com/protcode/isob; Mascot v2.2.07 (MatrixScience)
Quantitative determination of binding affinities: NITPIC, SEDPHAT v. 14.0, GUSSI (public domain)
Analytical size exclusion chromatography: GraphPad Prism Version 10 (Dotmatics, MA, USA)
Circular dichroism spectropolarimetry: Chirascan (Applied Photophysics, UK), GraphPad Prism Version 10 (Dotmatics, MA, USA)
Small Angle X-ray Scattering: ATSAS version 3.0.1
X-ray structure determination: CCP4 Program Suite v8.0.019, Phenix Program Suite v1.13, COOT v0.8.9, Refmac v5.8.0267

NMR spectroscopy: Topspin v4.0.6; CARA v1.9.0.b2; CCPNmr v2.4; SPARKY
Cryogenic-EM: Cryosparc; UCSF Chimera v1.13; UCSF ChimeraX v1.2.5, COOT v0.8.9; Phenix v1.13; ISOLDE v1.1.2; CheckMySequence (https://gitlab.com/gchojnowski/checkmysequence); MolProbity (Duke University), PDBePISA (Protein Data Bank in Europe)
Western blot analysis: FIJI app (RRID:SCR_002285)
Immunofluorescence staining: Imaris (Oxford Instruments, UK)
Transactivation assay: GraphPad Prism Version 10 (Dotmatics, MA, USA)
Statistics and Reproducibility: GraphPad Prism Version 10 (Dotmatics, MA, USA)

For manuscripts utilizing custom algorithms or software that are central to the research but not yet described in published literature, software must be made available to editors and reviewers. We strongly encourage code deposition in a community repository (e.g. GitHub). See the Nature Portfolio guidelines for submitting code & software for further information.

Data

Policy information about availability of data

All manuscripts must include a data availability statement. This statement should provide the following information, where applicable:

- Accession codes, unique identifiers, or web links for publicly available datasets
- A description of any restrictions on data availability
- For clinical datasets or third party data, please ensure that the statement adheres to our policy

Coordinates of the X-ray structure determined in this contribution have been deposited in the Protein Data Bank under accession codes 8ATI (https://doi.org/10.2210/pdb8ATI/pdb).
Chemical shifts of RAI2(WT, 303-465) were assigned and deposited in the BMRB under accession number 28085 (https://doi.org/10.13018/BMR28085).
Coordinates of the cryogenic EM structure have been deposited in the protein data bank under accession code 8ARI (https://doi.org/10.2210/pdb8ARI/pdb) and the density map has been deposited in the Electron Microscopy Data Bank (EMDB) under the accession code EMD-15603 (www.ebi.ac.uk/emdb/EMD-15603).
SAXS data have been deposited at the SASBDB server under accession codes: SASDQW5 (www.sasbdb.org/data/SASDQW5/), SASDQZ5 (www.sasbdb.org/data/SASDQZ5/), SASDQ26 (www.sasbdb.org/data/SASDQ26/), SASDQ36 (www.sasbdb.org/data/SASDQ36/), SASDQ46 (www.sasbdb.org/data/SASDQ46/), SASDQ56 (www.sasbdb.org/data/SASDQ56/), SASDQ66 (www.sasbdb.org/data/SASDQ66/), SASDQ76 (www.sasbdb.org/data/SASDQ76/), SASDQ86 (www.sasbdb.org/data/SASDQ86/), SASDQ96 (www.sasbdb.org/data/SASDQ96/), SASDQA6 (www.sasbdb.org/data/SASDQA6/), SASDQB6 (www.sasbdb.org/data/SASDQB6/), SASDQC6 (www.sasbdb.org/data/SASDQC6/), SASDQD6 (www.sasbdb.org/data/SASDQD6/), SASDQE6 (www.sasbdb.org/data/SASDQE6/), and SASDQF6 (www.sasbdb.org/data/SASDQF6/).
Source data are provided with this paper.

Research involving human participants, their data, or biological material

Policy information about studies with human participants or human data. See also policy information about sex, gender (identity/presentation), and sexual orientation and race, ethnicity and racism.

Reporting on sex and gender	All analyzed blood samples were collected from male individuals with metastatic prostate cancer. Prostate cancer affects only men.
Reporting on race, ethnicity, or other socially relevant groupings	Analyzing the biological relevance of the discovered molecular mechanism in subset of racial minorities was not within the scope of this research project. However, we consider that the study set-up will not create any blind spots in the anticipated study results and will not lead to disadvantage of any minority.
Population characteristics	In our study we have analyzed blood samples from prostate cancer patients of the Center of Oncology, University Medical Center Hamburg-Eppendorf, Germany. As we do not preselect any of these patients and the national healthcare system is generally accessible, we consider that our study represents an average of the population, selecting the Center of Oncology, University Medical Center Hamburg-Eppendorf, for treatment.
Recruitment	Patients were recruited during their routine visits for systemic treatment of metastatic prostate cancer at the University Medical Center Hamburg, Eppendorf between June 2018 and July 2022. The cohort reflects a typical population of patients with metastatic prostate cancer in Germany. Patients were included regardless of the number of previous therapies and the response to these treatment regimens. An ECOG0/1 status was required in order to avoid exposing patients to any major stress due to the additional amount of blood collected (which was taken as part of routine diagnostics). Beyond this, no further selection criteria were applied.
Ethics oversight	The study was approved by the ethical commission of the 'Hamburger Ärztekammer' (PV3779, PV5392). Patients provided informed consent for the participation in this study.

Note that full information on the approval of the study protocol must also be provided in the manuscript.

Field-specific reporting

Please select the one below that is the best fit for your research. If you are not sure, read the appropriate sections before making your selection.

☒ Life sciences ☐ Behavioural & social sciences ☐ Ecological, evolutionary & environmental sciences

For a reference copy of the document with all sections, see nature.com/documents/nr-reporting-summary-flat.pdf

Life sciences study design

All studies must disclose on these points even when the disclosure is negative.

Sample size	117. No sample size calculation for analysis of clinical samples was determined. The sample size used reflects the number of patients who agreed to participate during the patient recruitment period.
Data exclusions	No data was excluded from any analysis.
Replication	Unless mentioned otherwise, experiments were performed in biological replicates. Relevant information on statistical data analysis is provided in the legends of respective figure panels.
Randomization	In this observational analytical study, randomization is not applicable as no interventions or exposures were performed.
Blinding	In this observational study, blinding of patient samples was not possible because the samples were collected during a routine clinical visit and the treating physician already knew the patient's diagnosis. However, for further processing the samples were pseudonymized. Experiments were carried out without knowledge of personal or clinical data.

Reporting for specific materials, systems and methods

We require information from authors about some types of materials, experimental systems and methods used in many studies. Here, indicate whether each material, system or method listed is relevant to your study. If you are not sure if a list item applies to your research, read the appropriate section before selecting a response.

Materials & experimental systems

n/a	Involved in the study
☐	☒ Antibodies
☐	☒ Eukaryotic cell lines
☒	☐ Palaeontology and archaeology
☒	☐ Animals and other organisms
☒	☐ Clinical data
☒	☐ Dual use research of concern
☒	☐ Plants

Methods

n/a	Involved in the study
☒	☐ ChIP-seq
☒	☐ Flow cytometry
☒	☐ MRI-based neuroimaging

Antibodies

Antibodies used

Western Blot
RAI2 (clone D4W9P, #97857, 1:2000, Cell Signaling Technology, MA, USA)
CtBP1 (clone 3, #612042, 1:2000; BD Biosciences, NJ, USA)
CtBP2 (clone 16, #612044, 1:2000; BD Biosciences, NJ, USA)
CDKN1A (Clone 12D1, #2947, 1:2000; Cell Signaling Technology, MA, USA)
HSC70 (clone B-6, #sc-7298, 1:1000000; Santa Cruz Biotechnology, CA, USA)
HRP- (#7074; Cell Signaling Technology, MA, USA) or IRDye® (#925-32210, #926-68071; Li-Cor Biosciences, Germany) coupled secondary antibodies
Immunofluorescence staining
RAI2 (clone D4W9P, #97857, 1:250; Cell Signaling Technology, MA, USA)
CtBP1 (clone 3, #612042, 1:250; BD Biosciences, NJ, USA)
CtBP2 (clone 16, #612044, 1:250; BD Biosciences, NJ, USA)
EZH2 (D2C9, 1:125, Cell Signaling Technology, MA, USA)
H3K27me3 (C36B11, 1:250, Cell Signaling Technology, MA, USA)
Alexa Fluor 488 goat anti-rabbit IgG (H+L) (#A-11008, 1:300) and Alexa Fluor 546 goat anti-mouse IgG (H+L) (#A-11003, 1:300) (Life Technologies, CA, USA)
Chromatin Immunoprecipitation (ChIP)
CtBPs (#sc-17805, Santa Cruz, CA, US)
EZH2 (#C15410039, Diagenode, Belgium)
H3K27me3 (#C15410069, Diagenode, Belgium)
mouse IgG (#C15400001, Diagenode, Belgium) or rabbit IgG (#C15410206, Diagenode, Belgium)

Validation

Validation of the antibodies for use in immune-fluorescence experiments has been provided by the manufacturers except for the RAI2 antibody. For this antibody validation for immune-fluorescence staining was done in this study using genetically modified cell lines (Figures 2C, 4B).

Eukaryotic cell lines

Policy information about cell lines and Sex and Gender in Research

Cell line source(s)	VCaP (RRID:CVCL_2235, ATCC #RL-2876) and HEK293T (RRID:CVCL_0063, ATCC #CRL-3216) cells were provided by American Type Culture Collection (ATCC). KPL-1 (RRID:CVCL_2094, DSMZ #ACC-317) cells were provided by Deutsche Sammlung von Mikroorganismen und Zellkulturen (DSMZ).
Authentication	Tandem repeat profiling (Multiplexion, Heidelberg, Germany).
Mycoplasma contamination	Cell lines were monthly tested by the Vernor©GeM detection kit (Minerva Biolabs, Berlin, Germany). No mycoplasma contamination of any cell line was detected throughout the study.
Commonly misidentified lines (See ICLAC register)	None of the used cell lines is listed in the International Cell line Authentication committee register of misidentified cell lines.
